# Effectiveness of Aural-Oral Approach Based on Volubility of a Deaf Child with Late-Mapping Bilateral Cochlear Implants

**DOI:** 10.3390/audiolres11030035

**Published:** 2021-08-05

**Authors:** Paris Binos, Elena Theodorou, Thekla Elriz, Kostas Konstantopoulos

**Affiliations:** 1 Department of Rehabilitation Sciences, Cyprus University of Technology, Limassol 3036, Cyprus; eleni.theodorou@cut.ac.cy (E.T.); theklah07@gmail.com (T.E.); 2 Department of Speech and Language Therapy, University of Peloponnese, 24100 Kalamata, Greece; konstantok@hotmail.com

**Keywords:** aural approach, cochlear implant, volubility, protophones, speech analysis

## Abstract

Background: The purpose of this study was to investigate the effectiveness of aural-oral habilitation (AO) over the traditional speech-language therapy, based on the number of vocalization-volubility of a deaf child with late-mapping bilateral cochlear implants using sequential measurements. Methods: The spontaneous productions during child interactions were analyzed. The child (CY, 7;0 years old) with a mean unaided pure-tone average (PTA) hearing loss >80 dB HL was assessed by using an assessment battery. Study design consisted of two phases: (a) baseline (end of speech therapy) and (b) end of AO treatment. Protophones were analyzed via acoustical analysis using PRAAT software. Results: One-way repeated-measure ANOVAs were conducted within and between phases. The analyses revealed significant differences between the ‘phase’ and the vocalization outcome (F = 9.4, df = 1, *p* = 0.035). Post hoc analyses revealed the significant difference between the mean number of disyllable vocalizations of AO approach (*p* = 0.05). The mean number of vocalizations was calculated for each protophone type, but no other significant difference was measured. Conclusions: AO approach proved effective as measured through volubility. The outcome of this study is indicative and is a starting point for broader research.

## 1. Introduction

According to Cole and Flexer [1], the ear is the ‘doorway to the brain’. Even the smallest hearing deprivation can impede the acquisition of communication ability [2]. That is the purpose of cochlear implant technology. Cochlear implants (CI) can bypass hearing losses and provide the auditory neural pathways with the necessary speech stimulus. Cochlear implant is a medical device that uses electricity to stimulate the spiral ganglion cells of the auditory nerve to restore hearing loss. The purpose of this device is to convert sound to an electrical signal and deliver this to the hearing nerve, which bypasses the damaged hearing apparatus, specifically the inner ear. However, this is only half of the habilitation process, because, for a child to communicate effectively, a long postsurgical habilitation is needed. This period includes the implementation of a long and intensive educational evidence-based model.

### Communication Models

This period is designated by the communication model that parents must decide for their child during the extended postsurgical habilitation period, and after receiving counselling services from the interdisciplinary team. The aural-oral approach (AO) is not the only communication approach that parents could choose from, reinforcing the communication of their child with hearing loss. There are other approaches, which are:

(1) Visually based (sign, sign language, bilingual-bicultural and total communication);
(2) Auditory driven (among them, the aural-oral (AO) approach and auditory-verbal therapy (AVT));
(3) Traditional speech and language therapy [3].

There are findings suggesting that the visual communication approaches undermine speech and language development, and there is only a narrow window of time through which auditory stimulation could support the reorganization of cortical connections [4,5,6]. Geers et al. [7] published the most compelling support in cochlear implant literature, suggesting that parents’ use of sign language has no advantage either before or after CI. In fact, parental sign language use was associated with slower development of speech recognition and less intelligible speech in children of primary school age. On the other hand, children who enrolled in auditory speech-based approaches exhibited a statistically significant language advantage over those enrolled in sign language programs [8]. After all, a recent systematic review concluded that there is not enough ‘high-quality evidence to determine whether sign language in combination with oral language is more effective than oral language therapy alone’ [9] p.1. At the same time, intelligible speech in children with CIs has been associated with an oral-only approach [10].

As for auditory-driven approaches, their main aim is to reinforce the auditory cortex of the brain instead of relying solely on visual cues. Auditory-driven approaches take advantage of neural plasticity to develop brain as a listening brain, especially during the first three years of life. Auditory-driven approaches differ from the others in a number of ways. A difference to the traditional speech-language therapy approaches (SLT), which are not implemented in the auditory-verbal therapy principles, is the upgraded role of caregivers of the children. Parents here endeavor to incorporate the techniques and strategies of the auditory-driven approaches during everyday activities with their deaf child. The role of the caregivers is boosted for children who receive this kind of therapy and aims to develop spoken language skills [11]. The provision of auditory-verbal therapy (AVT) is undertaken by specialists who are extensively trained in these methods by the certifying body, the AG Bell Academy, or those who have undergone extensive post-graduate training to become listening and spoken language specialists. By contrast, the traditional speech-language therapy approaches are provided by clinicians who are certified speech-language therapists/pathologists through a traditional undergraduate curriculum or post-graduate programs that are not focused on promoting courses specialized in the technique approaches. Oral approaches are very different from traditional speech therapy. The aural/oral approaches are exclusively focused on children who are deaf and hard of hearing, while speech therapy covers a wide range of speech and language deficits or delays. The focus here is on rehabilitating children with comprehending or using verbal language to communicate.

There are differences between the traditional speech therapy but among the aural/oral approaches as well. Different methodologies have been developed within the frame of aural habilitation approaches because the applied methodologies and strategies are different. Despite the common basis, which is family’s decision to use spoken language, the auditory-oral (AO) approach encourages speechreading through the movements of mouth and body. By contrast, the evidence-based practice called ‘auditory-verbal therapy’ (AVT) aims at using only the child’s listening abilities to acquire spoken language. The child develops spoken language through one-on-one therapy and use of residual hearing with optimal amplification. This is a marked difference, and even some professionals are often confused about those systems. AO is also engaging parents during therapy but AVT does this to a much greater extent. AO is an approach that teaches a child to use his/her remaining hearing through amplification and the use of speechreading/natural gestures/visual cues to aid the child’s understanding of language. The use of any form of sign language communication is not encouraged. AVT is finally applied by certified professionals with extensive training and knowledge of the ten (specific) AVT principles, while AO requires professionals who are qualified only in speech-language pathology or education [11].

Several studies have investigated the efficacy of AO approach for children with CI. Montag and her colleagues [10] suggested that the application of the AO approach is beneficial for children with CI in terms of speech intelligibility. Other studies highlighted that there are better language outcomes when communication is based on auditory-oral approaches [12,13,14]. In addition, it was found that AO promotes the onset of canonical syllables and supports the phonemic recognition [3]. Both the onset of canonical syllables and phonemic recognition can predict the vocabulary size. However, there are no studies that go a step backward to investigate the first productions (protophones) of CI children. Therefore, the study presented here aims to address this gap.

The current study is the first part of a larger-scale study, investigating the relative efficacy of the aural/oral (AO) approach in daily clinical practice as compared to the traditional speech-language therapy in a child with cochlear implants. Toward this, it reports on the spontaneous productions (protophones) of a bilateral cochlear-implanted boy during parent/specialist interactions, focusing on the vocalization of canonical babbling. This dyadic vocal interaction increased the rate of protophone vocalizations and especially the forms of canonical babbling [15,16].

Based on this framework, the babbling period is closely related to future speech development, since it establishes a possible prognosis for subsequent speech development. According to Oller et al. [17] p.223, ‘delayed onset of canonical babbling can predict delay in the onset of speech production’. This babbling stage is also recorded in typically developing infants, starting at 6–7 months of age [18]. The infants produced the majority of their protophones across their first year of life [19]. Schauwers et al. [20] provided more evidence that the onset of babbling can be accounted as a precursor to speech as well as a speech motor control milestone. Despite the importance of this stage, there is a scarcity of studies focused on prelinguistic vocal development with hearing impairment [21].

## 2. Case Presentation

The aim of this study was dual:

(1) To compare the efficacy of the AO approach and traditional speech-language therapy based on the prelinguistic vocal development;
(2) To describe the canonical babbling structures that an implanted child uses.

In speech-language therapy, there are various levels of evidence. Each of these levels incorporates a plethora of types from randomized controlled trials (RCTs) and meta-analyses to expert opinions. All types of research design have advantages and disadvantages. Among them, the value of small-scale research is well documented in the literature, and clinical practice is mainly focused on small-scale research for many reasons related to time and resources [22]. Even though case studies do not constitute the highest level of evidence, they do provide significant support for clinical practice in speech and language therapy. Regarding this, the literature on studies of prelinguistic vocal development in children with cochlear implants is mainly based on case studies [23]. In a parallel field, in the augmentative and alternative communication literature, most of the evidence is based on single-case studies [24]. Moreover, case studies provide a detailed depiction of important individual differences instead of average effects, as RCTs do [25].

Currently, smaller samples have a meaningful role, especially during the evaluation of approaches that need more data to support their evidence. This is also the trend moving from the restrictive evidence-based approaches (EBPs) to additional forms that are more inclusive. This inclusive evidence, mentioned as evidence-informed practices (EIPs), has involved expert opinions and case studies [26].

The chosen experimental design is suitable for a study aiming to measure the efficacy of an intervention based on a small number of participants and, more specifically, the single-case experimental design [27]. Therefore, the present study research design was based on a single person whose performance was measured over time, which included sequential recordings and involved the application of the two therapy approaches sequentially.

The present AB design was carefully developed before the AO intervention was conducted in a university rehabilitation setting, including repeated measurements, specific data analysis and statistics. This AB design is not a case report, since the performance of the participant was repeatedly measured in the absence and presence of AO approach.

Therefore, there were two phases:

(A) Baseline-traditional speech therapy (end of speech therapy);

(B) End of the AO treatment phase after a year of treatment.

The comparison between the two approaches was based on the number of vocalizations (volubility) during the canonical babbling stage.

### 2.1. Participant

The participant was a congenitally hearing-impaired boy, CY, with bilateral cochlear implants and Greek-Cypriot speaking and hearing parents. The family of the boy was characterized as medium socio-economic class. Before the habilitation program began, the family gave written consent for their child’s participation, according to the ethical standards set for the confidential and anonymous treatment of the participant’s data. CY had no other experience of any kind of therapy before the first visit at the University Rehabilitation Clinic in 2018. Table 1 depicts all characteristics with respect to hearing loss.

#### Reason of Child Selection

CY was selected because he received the first cochlear implant (right ear-CI_R_) at the chronological age of 7 months, while the external component was placed after two years of age. The boy received another cochlear implant on the left side, at the chronological age of 3;7 years (CI_L_). CY came at the University Rehabilitation Clinic in September 2018 (7;0 years old) with a referral from an ENT department, for a full speech-language assessment aiming to participate in an intervention focused on the development of speech and language production and comprehension. CY was wearing both CIs for more than 10 h per day during the implementation of each approach, and the fitting process was based on a clinic visit each 6 months in the post-surgery period. The AO habilitation program of the clinic used auditory-verbal techniques to train hearing-impaired children with cochlear implants. CY had no other disabilities and had unknown deafness etiology wearing cochlear implants (type Nucleus-24 of Cochlear Co) to both ears. Prior to implantation, the boy had average unaided hearing loss of more than 90 dB HL on both ears (pure tone average >80 dBHL).

The boy was assessed by using the clinic’s assessment protocol used in University Rehabilitation Clinic. An attempt was made to evaluate the child’s verbal ability using two tasks of the Diagnostic Test of Verbal Intelligence (DVIQ) [28]. The test evaluates the productive vocabulary, vocabulary comprehension, the comprehension of morphosyntactic structures (sentences) and sentence repetitions. For the comprehension of sentences, the child had to select a picture (out of three) that corresponded to a given utterance, and, for the assessment of verbal production, the child had to name an item depicted on one page; however, the child failed to respond in all the above-mentioned tasks. However, CY was also evaluated by a language-free test, named TONI-4 (6;0–89;11 years old) [29], in order to exclude non-verbal intelligence deficits; the outcome of TONI-4 classified the boy as ‘average’ (scored as 7;3 years old, typical) based on his chronological age.

CY received a full oral-motor examination as well. Nothing pathological was noted during oral screening examination. His speech-language assessment classified his verbal skills at the same level with an infant during the first year of language development based on the analysis of spontaneous speech. His present auditory skills were also evaluated based on a criterion-referenced questionnaire titled ‘CY-Meaningful Auditory Integration Scale-MAIS’. The administration of this questionnaire gave 28/40 score at the beginning of the SLT approach. Important milestones of language development were very poor such as the onset of reduplicated babbling. At the beginning of speech therapy, the boy had had 4;5 years of post-implant experience already, but his communication was heavily dependent on hand cues, and only some isolated nuclei or monosyllables, structured with consonant-like onsets, were produced, based on the analysis of spontaneous speech. Overall, his communication profile was characterized by hesitation, long pauses, poor management of prosody and unintelligible speech.

### 2.2. Procedure

The procedure was carefully designed prior to the implementation of the intervention-target (AO approach). Based on the current AB design, one attempt consisting of three longitudinal periods was recorded to demonstrate the efficacy of AO in comparison with the traditional speech-language therapy. In order to demonstrate AO efficacy, a comparison between the two approaches was conducted after a year of implementation of each of the habilitation programs (A, B). CY joined the first phase of the speech-language therapy (chronological age 7;0, post-implantation age 4;5) program, and he left the program after a year of intervention. The next phase began (chronological age 8;0, post-implantation age 5;5) with the AO intervention program, and ended a year later.

Protophones were recorded by using a SONY-D50 portable linear digital recorder with a sampling rate of 44.1 KHz and 16-bit precision. This recorder uses two built-in electret condenser microphones covering a wide sound range with a natural sounding image. Two audio recordings of 45 min per session each week aimed to capture spontaneous interactions over a span of the last three months of each intervention. Therefore, 360 min per month were analyzed for each intervention over this three-month period.

Subsequent editing was performed to remove all vegetative data and productions masked by external noise or sounds. Sounds such as laughing, sneezing, hiccoughing, burping or long pauses were classified as vegetative sounds or fixed signals and were excluded. These criteria yielded for all recordings during the last three months of comparison after the implementation of each habilitation approach. As a result, only the isolated vocalizations (protophones) were analyzed involving rapid combinations of consonants and vowels. Acoustical analysis was based on inspection records using PRAAT analysis software for Windows (4.110).

### 2.3. Data Analysis

Precanonical protophones cannot be transcribed using the International Phonetic Alphabet (IPA), since they do not shape well-formed consonants and vowels [19]. On the other hand, the phonological characteristics of canonical babbling were very similar to early words with respect to syllable types and shapes [30,31]. Canonical babble is shaped by a rapid transition (<120 ms) between consonant-like and vowel-like portions of the syllable [32].

The current methodology was based on Oller’s [33] infraphonological theory for the classification of protophones. The recorded and analyzed protophones were classified into three categories, concerning the chronological age of the participant, and are listed below:

(1) Monosyllables (CV): According the infraphonological methodology, the presence of a full vowel-like element as well as one consonant-like element constitutes a canonical syllable, only if there is a rapid transition between them at the same time.
(2) Disyllables (VCV, CVCV): canonical, superior formational structures.
(3) Trisyllables (CVCVCV).

### 2.4. The Implementation of the Auditory-Oral Approach

The implementation of the AO approach permits speechreading but excludes the use of sign language. On the other hand, only principles and techniques that foster listening and spoken language of auditory-verbal therapy (AVT) were followed and mentioned bellow. Each week, S.M.A.R.T goals (Specific, Measurable, Achievable, Realistic, Time-Bound) were settled while parents engaged in sessions to practice these techniques in order to use them in everyday situations. Each session started with the Ling six-sound test [34,35] because it can provide useful information, as does pure tone stimuli, during the detection level [36]. Our individualized therapy plan included goals for the enhancement of auditory training based on Erber’s [37] hierarchy of listening skills. Four stages shaped this training: detection, discrimination, identification and comprehension.

The components of our AO program involved the implementation of many techniques that aimed to develop listening and spoken language skills in terms of production and comprehension. These techniques were derived from the AVT approach and were enriched with the simultaneous use of speechreading. These techniques are steps designed to achieve the goals that were set and placed the emphasis on learning as the building block to learning and acquiring spoken language. These techniques included: speaking to the child at a near distance for improved audibility, positioning the therapist behind or next to the child in order to discourage speechreading, acoustic highlighting and auditory bombardment [38,39]. The list of expressions that were used by the researcher includes commands and prompts, which are found in the relevant literature [11], such as ‘audition first’, ‘point out sound and name it’, ‘keep the serve and return going’, ‘describe actions and thoughts’, ‘make it easier to listen’, ‘expect an answer’, ‘expand child’s utterances’, ‘what did you hear’? and ‘create an auditory sandwich’.

## 3. Results

**Figure 1:**
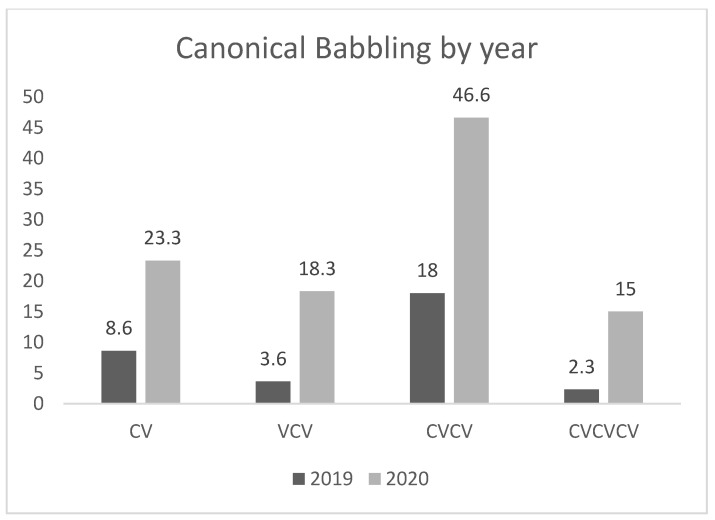
Mean volubility of the last three months of each intervention.

Audio recordings of 90 min per week (two sessions of 45 min) over a span of the last three months of each intervention approach were analyzed. Figure 1 shows the mean number of vocalizations for each protophone type of the last three months for each habilitation approach. As can been seen, there is an overall improvement in volubility after the implementation of the second habilitation approach AO. Cut-off levels of significance were set at 0.05 for all tests.

Post hoc analyses of the data revealed a significant difference between the mean number of disyllable vocalizations of approach B (AO approach) in comparison with approach A (traditional speech therapy) (*p* = 0.05). Post hoc analyses did not reveal any other significant difference, despite the observed graphic improvement of the mean number of vocalizations per type between the two habilitation approaches.

As shown in Table 2, one-way repeated-measure ANOVAs were conducted within and between subjects to assess whether there were statistical differences in the primary outcome measure with regard to baseline (approach A) and therapy AO (approach B). The mean number of vocalizations was calculated for each protophone type. Specifically, variable ‘phase’ was significantly related to the vocalization outcome (F = 9.4, df = 1, *p* = 0.035).

Because of a possible violation of the sphericity assumption, we used the Greenhouse–Geisser (G–G) adjusted univariate tests where necessary (*p* > 0.05). The Levene Test of Equality of Variance was carried out for the variable ‘type’ of vocalization (Table 3). The equality of variances test revealed that equality of variances was not violated. Due to the small sample, Tukey post hoc analyses were implemented. Table 4 depicts Tukey post hoc comparisons between the two habilitation approaches and between the ‘type’ of vocalization and the variable ‘phase’. The effect of the AO approach was significantly better regarding the mean number of vocalizations (*p* < 0.05), in comparison with the traditional speech-language therapy.

## 4. Discussion

This longitudinal study was about spoken language and intervention. The aim of the present study was to explore the effect of AO on the rate of language growth, in terms of phonetic and phonological development, for a young child with hearing loss. The aforementioned data illustrate the positive efficacy of the Aural-Oral (AO) approach on volubility of a young bilateral cochlear implant recipient who later received the external part of the CI, in contrast with the traditional speech-therapy approaches.

The present study commits new data of AO efficacy with findings based on canonical babbling stage and the volubility of a young CI recipient. The present findings are in agreement with the study of Geers et al. [7] because the verbal development is built by the spoken language input of the AO approach, instead of the traditional speech-therapy approaches. Indeed, there is an extensive work in the literature supporting even better language outcomes when communication is based on auditory-oral approaches [12,13,14]. Speech intelligibility scores in long-term sample of CI users seemed to be benefited by the higher reliance on auditory-oral communication [10].

There is a lack of studies focusing on cochlear implantation as speech production aids because the majority of the research is about speech reception and perception skills following implantation. The speech-produced outcomes by prelingually deaf children are far less studied [40]. Moreover, children such as our participant, CY, with greater residual hearing before implantation (pure tone average >80 dBHL), are more likely to be placed in auditory-driven programs [41].

Literature presented so far compares between visual (sign language, total communication and bilingual-bicultural) and auditory-driven approaches (AO or auditory-verbal therapy) [42,43,44]. The present case study differs since it adds new findings for the efficacy of AO habilitation approach over the traditional speech-therapy approaches. This investigation of AO efficacy is the first at the pre-linguistic level. The AO habilitation approach seems to handle better the sensitive period for auditory stimulation against the speech therapy techniques, even in a late CI recipient. This is also in agreement with the findings of studies that focus on the neural plasticity and the re-organization in children with cochlear implants, which mention the necessity for an immediate access to auditory stimulation approaches [45].

The current design excluded control biases because the patient was tested longitudinally, and there were no other events that occurred concurrently during the implementation of any intervention. On the other hand, the efficacy of the AO approach should not be overinterpreted, since it cannot be assumed that all children with CI would be expected to respond similarly. Despite this weakness, the present systematic findings offer a statistically significant result for AO efficacy, monitoring the progress in volubility, as measured by the number of vocalizations, of a young child with bilateral cochlear implants.

## 5. Conclusions

The study presented here aimed to shed some light on the effectiveness of the AO approach, even for the pre-linguistic period, for the verbal development of a bilateral cochlear-implanted child. The results indicate the efficacy of the AO habilitation approach on volubility as an important developmental marker. Indeed, further investigation is required in order to corroborate the results. Overall, the outcome of this foundation study is indicative and is a starting point for more research.

Nonetheless, the significance of this study, in terms of clinical application, cannot be underestimated. The early suggestion is that speech and language therapists can apply the AO approach for implanted children aiming to first promote volubility and subsequently speech and language development.

## Figures and Tables

**Table 1:** Characteristics of the single-case study involved in this study.

**Child/Birth**	**Age of Receiving CI**	**Age of Functional CI**	**SLT**	**AO**
CY	CI-Right 0:7	CI-Right 2:7	2018-19	2019-20
8 August 2011	CI-Left 3:7			
**PIA (start)**	**Reason for CI**	**Onset of HL**	**Diagnosis**	**Additional Disability**
PIASLT 4:5 (CA 7:00)	Profound HL	Congenital	S/N	No
PIAA0 5:5 (CA 8:00)				

**Table 2:** Effect of AO (approach B) on volubility versus traditional speech-language therapy (approach A). Repeated measures ANOVA.

**Within Subjects’ Effects**					
	**Sum of Squares**	**df**	**Mean Square**	**F**	***p***
RM Factor 1	1765	3	591.0	14.53	<0 .001
RM Factor 1 Phase	180	3	60.3	1.49	0.276
Residual	495	12	42.9		
Note. Type 3 Sums of Squares					
**Between Subjects’ Effects**					
	**Sum of Squares**	**df**	**Mean Square**	**F**	***p***
Phase	1457	1	1440	9.41	0.035
Residual	607	4	155		
Note. Type 3 Sums of Squares Assumptions					
**Tests of Sphericity**					
		**Mauchly’s W**	***p***	**Greenhouse–Geisser ε**	**Huynh-Feldt ε**
RM Factor 1		0.0832	0.274	0.485	0.666

**Table 3:** Levene’s (homogeneity of variances) of the “type” of vocalization (protophones).

Equality of Variances Test (Levene’s)				
	F	df1	df2	*p*
CV	5.468	1	4	0.085
VCV	0.339	1	4	0.6
CVCV	1.73	1	4	0.261
CVCVCV	12.493	1	4	0.021

**Table 4:** Tukey post hoc comparisons between habilitation approaches and “type” of vocalization per phase.

**Post Hoc Comparisons–Phase**								
**Comparison**								
**Phase A**		**Phase B**	**Mean Difference**		**SE**	**df**	**t**	**p_tukey_**
2019	-	2020	−15.9		5.27	4.05	−3.11	0.031
**Post Hoc Comparisons–RM Factor 1 Phase**								
**Comparison**								
**RM Factor 1**	**Phase A**	**RM Factor 1**	**Phase B**	**Mean Difference**	**SE**	**df**	**t**	**p_tukey_**
CV	2019	CV	2020	−11.575	6.88	10.8	−1.699	0.694
VCV	2019	VCV	2020	−11.887	6.88	10.8	−1.739	0.67
CVCV	2019	CVCV	2020	−24.977	6.88	10.8	−3.555	0.05
CVCVCV	2019	CVCVCV	2020	−13.950	6.88	10.8	−2.050	0.447

## Data Availability

Not applicable.
